# Cumulative Dynamics of Independent Information Spreading Behaviour: A Physical Perspective

**DOI:** 10.1038/s41598-017-05899-5

**Published:** 2017-07-17

**Authors:** Cangqi Zhou, Qianchuan Zhao, Wenbo Lu

**Affiliations:** 1 Center for Intelligent and Networked Systems (CFINS), Department of Automation, Tsinghua University, Beijing, 100084 China; 2 Tsinghua National Laboratory for Information Science and Technology (TNList), Tsinghua University, Beijing, 100084 China

## Abstract

The popularization of information spreading in online social networks facilitates daily communication among people. Although much work has been done to study the effect of interactions among people on spreading, there is less work that considers the pattern of spreading behaviour when people independently make their decisions. By comparing microblogging, an important medium for information spreading, with the disordered spin glass system, we find that there exist interesting corresponding relationships between them. And the effect of aging can be observed in both systems. Based on the analogy with the Trap Model of spin glasses, we derive a model with a unified power-function form for the growth of independent spreading activities. Our model takes several key factors into consideration, including memory effect, the dynamics of human interest, and the fact that older messages are more difficult to discover. We validate our model by a real-world microblogging data set. Our work indicates that, other than various features, some invariable rules should be considered during spreading prediction. This work also contributes a useful methodology for studying human dynamics.

## Introduction

Information spreading is of great significance to the communication in people’s daily life, the marketing strategies of corporations, and the concentration of public opinion^1^. To investigate how individuals respond to received information, most existing research studied the interactions among them. However, to our best knowledge, how individuals *independently* decide whether or not to spread information has not been well answered yet. In this paper, we study cumulative dynamics of independent spreading behaviour by probing retweeting, one of the most typical information spreading processes in online social networks. Our target problem is to explore if there exists a model with a simple unified form that governs the growth of all independent retweeting activities in our data set.

Retweeting is the behaviour of copying received messages and reposting them on microblogging platforms. The decision of retweeting could be made under the influence of others or, independently. It is intuitive that there exist certain patterns of interactions among people, such as the cascading effect^2–4^, the co-existence of competition and cooperation^5^. However, most existing studies, whether theoretical^6–8^ or empirical^9–11^, do not discriminate the independence property of spreading activities from others, although it makes up a large portion of all spreading activities. Therefore, rather than focusing on the hierarchical structure, we focus on the retwitters who directly retweet initialized messages from influentials, without any influence from others. The reason we only consider influentials is that there is hardly any information about spreading dynamics if a message is retweeted by only a few retwitters, though we do not make any particular assumptions about influentials in our model derivation. One of the benefits of studying direct retwitters is that, due to the filtering of direct retwitters, networks can be reduced to a much smaller scale. Meanwhile, this filtering process does not reduce the representativeness of retweeting activities, since that a large number of spreading processes originated from influential users end up within their one-degree follower networks^11^.

Specifically, through empirical data, we trace the reposted messages (retweets) of popular initialized/original messages (tweets) by direct retwitters in Sina Weibo, China’s most popular microblogging service. Direct retwitters can be identified by text parsing utilities. Then, similar to other studies^12–14^, the change of the number of retweets allows us to investigate the dynamics of independent spreading behaviour. We will investigate whether there is a model with a unified form that applies to all responding activities.

Of the existing explanatory models of information spreading, some intend to explain the microscopic interactions among individuals^15^, some intend to characterize the resulting effect^12, 16^, and some bridge the gap between microscopic mechanisms and macroscopic phenomena^2, 4, 5^. We aim to come up with a modeling methodology that is able to derive a mathematical model for phenomenal results based on some intuitive microscopic conjectures. To achieve this goal, we try to model the growth of retweets by the analogy with spin glass models. The motivations of this methodology are two-fold. The first one is the inspiration from the works of Johansen and co-workers^14, 17, 18^, who reported several experiments on the Internet which could be explained by the models similar to the *Trap Model* of spin glasses. The second motivation is the realization that aging effect is likely to exist in the growth of retweeting activities, since it is harder to discover older messages in microblogging. To derive our model, we investigate the *Ising Model*, the *Random Energy Model* ^19^ for microscopic mechanisms and the definition of ground state of spin glass systems. Then we investigate the Trap Model for the explanation of the aging effect in spin glasses. We identify several corresponding relationships between the retweeting behaviour of microblogging users after the publication of tweets from influentials, and the relaxation responses of spin glasses after the switch off of magnetic fields at low temperature. Based on some intuitive conjectures, we derive a power-function model to describe the change of cumulative number of retweets over time. These conjectures, such as memory effect, the dynamics of human interest, and the fact that it gets more difficult to discover older messages, are the key elements in our modeling process. And they are demonstrated, to some extent, by empirical experiments or relevant references. Then we fit our data by the derived model. The results show that although the content of messages and the influence of original publishers are diverse, our model fits well to most of our empirical data. We also show the predictability of our model.

Our work contributes a useful methodology, the analogy with physical systems, for studying human dynamics. The discovered rule, that applies to the growth of different retweeting activities with a unified form, reveals the nature of complexity in retweeting activities. We hope that our work will shed some light on the study of human dynamics^20–24^. Our work also indicates that, other than various features adopted in well-tuned machine learning models, some invariable rules, such as the power-law growth of independent retweeting activities, the memory effect in human behaviour, should be taken into consideration during the prediction of information spreading.

## Results

In this section, we introduce the whole modeling process. The section is outlined as follows: At first, to obtain a set of independent retwitters, data preparing and preprocessing are carried out. Then we derive the model for the growth of retweets by the analogy with relevant spin glass models. Next, we fit empirical data with the derived model. Then, we carry out several experiments to validate our model by real data from Sina Weibo. At last, we show the predictability of the derived model.

### Data Preparation and Preprocessing

For this study, we obtained two data sets, a message set and a link set. The message set allows us to trace every retweet and its corresponding retwitter of an original tweet. The link set describes the *following* relationships among users. Thus, we are allowed to locate every single retweeting activity on real-world network structures.

**Figure 1:**
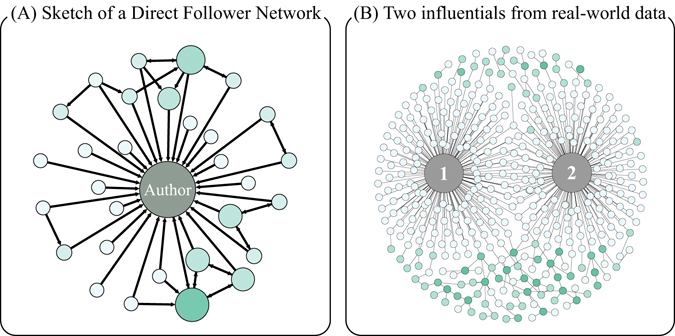
An illustration of Direct Follower Networks. (**A**) An influential user lies at the center of a DFN, and every other user follows him. The influential is mainly an *author* of popular tweets. All the followers of the influential and all the links among these followers are included in the DFN. (**B**) We show the structure of DFNs retrieved from Sina Weibo. A tweet is passed on from one influential to another. The overlap of the two DFNs is the bridge for tweet spreading. We sample the original data of the two DFNs for a better illustration. Nonetheless, the main structure has been retained.

A tweet could be spread to distant users from its original publisher. Since that all the followers of current retwitters could receive the message and expose it to their own descendants, the entire network structure, on which the original tweet spreads, might be too large to study. Goel *et al*.^11^ found that a vast majority of spreading cascades terminate within one degree of an initial seed. We further defined *Direct Follower Networks* (DFNs) in our former paper^25^ to describe the spreading activities happened within the first-layer of followers of an influential user. Figure 1 shows a schematic diagram of a DFN and two overlapped DFNs from real data. The formal definition of DFNs is as follows,

**Definition 1** *A Direct Follower Network associated with an influential user u, is a directed graph with a node set* ${\mathcal F}$ and a link set ${\mathcal L}$. The set ${\mathcal F}$ consists of all the followers of *u* with distance 1. The set ${\mathcal L}$ consists of all the directed links among the nodes in ${\mathcal F}$.

**Figure 2:**
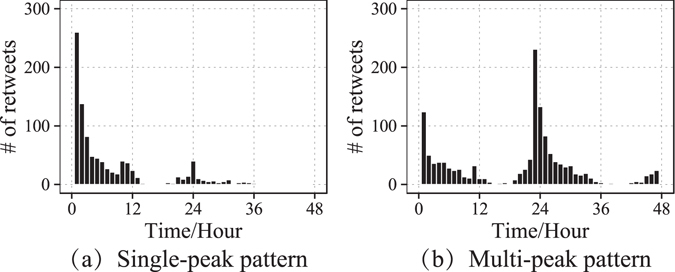
Single-peak and multi-peak patterns of retweeting intensity.

On this simplified network structure, we trace the retweets of 3,506 original tweets initialized by the top 10 most active (in the sense of the amount of initialized tweets) influentials in our data set. Then we calculate the ratios of the retweeting activities on DFNs to the total amounts of retweets. The results show that the ratios exceed 80% for nearly 90% of original tweets, which means the overwhelming majority of retweeting activities happen on DFNs. In addition, as shown in Fig. 2, the hourly intensity of retweeting activities can be roughly classified into single-peak and multi-peak patterns. Multi-peak patterns involve more complicated factors than single-peak patterns. These multi-peak patterns account for less than 5% of all samples in our data set. Therefore, they are removed in the following analyses.

**Figure 3:**
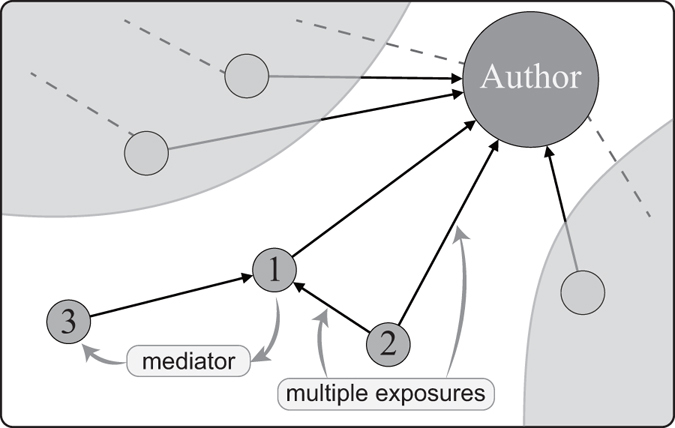
A schematic diagram of direct and indirect retwitters. After the publication of an original tweet from the author, direct followers 1 and 2 will immediately receive the message. Suppose 1 reposts the message, he becomes a direct retwitter. As a consequence, on the one hand, he will be the mediator for his follower 3, a possible indirect retwitter. On the other hand, because of the existence of the direct link from 2 to 1, the retweeting behaviour of retwitter 1 increases the number follower 2 receives the message.

After the simplification of network structure, we discuss the independence among retwitters. A retwitter could directly retweet a message after receiving the message exactly once from the author, and he also could receive the message multiple times from other retwitters and then retweet it. The retwitters in the former case, who are reckoned as *direct* retwitters, are considered as independent retwitters with each other because their decisions of retweeting are not affected by other retwitters. Figure 3 demonstrates direct and indirect retwitters. The so-called triad closure makes multiple exposures happen. The effect of multiple exposures on information diffusion in social networks is non-trivial. It is of great importance for researchers from a broad range of fields^26, 27^. In our data set, the ratios of the numbers of direct retwitters to the numbers of retwitters in DFNs exceed 70% for more than 90% of all tweet samples, which means the overwhelming majority of retwitters in DFNs are direct retwitters.

**Figure 4:**
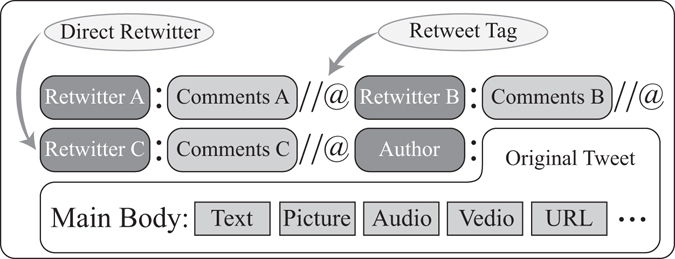
Locating the direct retwitter in the text of a retweet. Retwitters C, B and A retweet the author’s original tweet chronologically. Retwitter C is the closest to the author. Thus, C is reckoned as a direct retwitter.

We identify these direct retwitters by text parsing utilities such as regular expressions. Specifically, among all the retwitters, every direct retwitter lies on the structure of DFNs, since other retwitters can only see the original message through some mediators. As shown in Fig. 4, we identify direct retwitters by parsing the retweet tag in the text of retweets, and locating the positions of retwitters to the author. Admittedly, it is possible that a user could bypass his mediators and “directly” reposts a message, even though he saw the message from mediators. We ignore this situation since it is a much less convenient way for retweeting in microblogging. After the filtering and parsing processes mentioned above, we obtain our data set for modelling independent retweeting behaviour.

### Model Derivation

We first introduce the motivations of modeling retweeting behaviour by the analogy with spin glass models. Then we describe the connections between them in detail. At last, we give the mathematical details of our model.

The motivations that we try to model the growth of retweets by the analogy with spin glass models are two-fold. The first one is the inspiration from the works of Johansen and the co-workers^14, 17, 18^, who reported several experiments that characterize the dynamical responses of Internet users to some bursty events. One of their experiments is to probe the downloading activities of a scientific paper after the publication of a related online interview. The relaxation dynamics to the bursty events is explained as a barrier crossing in the Trap Model^28^ of spin glasses. The second motivation is the realization that aging effect is likely to exist in the growth of retweeting activities. The spin glass model we make analogies to are aimed to give reasonable interpretations for aging, which represents the slow magnetic relaxation dynamics existed at all time scale in spin glass materials. A typical feature of aging is that, the longer the waiting time, the slower the relaxation. In our Weibo systems, messages are ordered chronologically. The older a message is, the harder it is to find for retweeting. Hence, the growth of retweets shows a similar behaviour as the aging effect of spin glass.

Here we give a brief description of the spin glass models related to our work. A spin glass is a disordered system. Several intermetallic alloys and insulating compounds are made as spin glass experimental samples. These materials show some common behaviours, such as aging. The *Ising Model* is used to describe a set of interacting (*p*-)spins on lattice^19^. The minima model assumes that the spins can only take on the values 1 or −1. A *configuration* of a system, which assumes to be comprised of *N* spins, is an assignment of certain values to each spin. The system consists of ${2}^{N}$ configurations. Based on the Ising Model, the *Random Energy Model* (REM)^19^ assumes that, (1) the energy levels *E*_*i*_ corresponding to each configuration are independent and identically distributed normal random variables; (2) the probability that a system is in a certain configuration is proportional to $\exp (-{E}_{i}/T)$, where *T* represents thermodynamic temperature. The solution to the model indicates that an exponentially large number of configurations are distributed on a concentrated *ground state*. The probability that a system is on other states is almost zero.

Based on the REM, the *Trap Model* ^28^ characterizes the effect of aging in the magnetic relaxation processes of spin glasses. The model assumes that a metallic spin-glass instance can be decomposed into a lot independent spin glass subsystems. The energy state of a subsystem is characterized by its own configurations. And the energy *landscape* of the whole system is rough and hierarchical. The reference energy *f*_0_, which is specified by the ground state revealed by REM, forms a “plane” on the landscape. There are many local minima, which trap the subsystems into metastable configurations, on that plane. The depth of these traps is exponentially distributed with lower probability for deeper traps. And the deeper the trap is, the longer a subsystem stays in it. A transition between two configurations that are on the plane is almost instantaneous since that there is no energy difference between the configurations. Hence, most of the time, subsystems are trapped in these pits with relatively lower energy levels. A subsystem needs extra energy to escape a trap, and transits to another energy level. However, due to the constraint of structure, a transition of a subsystem between configurations occurs only between adjacent traps. It is unlikely that a subsystem can randomly transit to any trap on the plane. The ensemble of the transitions of all subsystems leads to the magnetic relaxation of the whole system. The model also introduces an exponential decaying factor for the convergence of integral.

A Weibo system, which is characterized by a Direct Follower Network of an author, consists of many independent retwitters. Each follower corresponds to a subsystem in spin glasses. The ground energy level of a retwitter corresponds to the least active state of the retwitter. People carry out different tasks in their daily lives. Once a person is dealing with a task, he is “trapped” by it. Since he is now more active compared to the ground energy state, the energy landscape formed by these tasks is filled with humps rather than pits. The difficulty and complexity to accomplish these tasks can be different. More difficult tasks will cost people longer time to deal with. And the property associated with the difficulty of tasks are assumed to be exponentially distributed. And this distribution in our Weibo systems will be demonstrated later. Now an energy landscape similar to that of spin glasses has been established. Analogous to spin glass relaxation, the ensemble of the transitions of retwitters among the humps created by original tweets, leads to the growth of the number of retweets.

We introduce two important facts in retweeting behaviour. The first is the *memory effect*, which characterizes the correlations among a series of tasks. Intuitively, the effect implies that people will deal with tasks in a logical order, and will not randomly transit between tasks. There are a lot of evidence that human activities show memory effect, i.e., they are not Markovian^29^. Considering the REM, adjacent system configurations on a grid are only different in the values of a few particles. A system can only transit to its currently adjacent states. And it limits large variations in energy levels. The second conjecture characterizes the bursty nature of the dynamics of human interest^20^. One of the basic properties is that the longer the time a person is doing one thing, the more likely he will lose his interest and move onto another task^21^.

The main corresponding relationships between spin glasses and Weibo systems are summarized in Table 1.

**Table 1:** Corresponding relationships between spin glasses and Weibo systems.

Item	Description
Individual Correspondence	A spin glass subsystem corresponds to a direct retwitter of an influential.
Individual Independence	Direct retwitters are independent with each other. The independence property appears in REM when *p* becomes large, where *p* is the number of spin interactions in the Hamiltonian.
Magnetic Field Correspondence	The external magnetic filed applied to a disordered system corresponds to the publication of a tweet by an influential. This publication can be viewed as a “field” applied immediately to the DFN of the influential.
State Transition Correspondence	The state transitions among energy states of spin glass subsystems corresponds to the transitions from different humps created by different tasks in people’s daily life.
Relaxation Correspondence	The magnetization relaxation of a disordered system corresponds to the spreading saturation of an original tweet in Weibo systems.

The main conjectures in spin glass systems also correspond to the conjectures in Weibo systems. They are, the exponentially distributed landscape, memory effect and the dynamics of human interest. These conjectures are demonstrated, to some extent, by either empirical experiments or relevant references.

Next, we give the mathematical details of model derivation. The configurations in REM correspond to different states when an individual is dealing with different tasks. Intuitively, more difficult tasks will cost people longer time to deal with. For instance, in Weibo systems, we will spend different lengths of time for messages with different contents. Let *f* denote the property associated with a task that will affect the length of time when people deal with it. The property could be associated with the complexity of mathematical problems, or the attractiveness of tweets. The distribution of *f* is assumed to be exponential, which is

1 $$P(f)=\frac{x}{T}\exp \frac{x({f}_{0}-f)}{T},\quad f\in [{f}_{0},{\rm{\infty }})$$

where *f*_0_ is the reference state in which people stay in their normal status of activeness. *f*_0_ corresponds to the ground state derived in the REM. The difference with the Trap Model is that *f*_0_ is our lowest energy state, and *f* is always equal or greater than *f*. The exponential form of the distribution will be demonstrated later by our Weibo data set.

The landscape is filled with humps, which means that people will transfer from ground state to more active states when he deals with some tasks. The expectation of *f* is

2 $$\langle f\rangle ={f}_{0}+\frac{T}{x}$$

*Parameter T* corresponds to the activeness of a user. The more active he is, the more probable he will be to deal with complex tasks. *Parameter x* corresponds to how simple, on average, users’ tasks are. The simpler the tasks, the lower the humps on the landscape.

According to the Arrhenius Law, the time for dealing with a task with property *f* is,

3 $$\tau ={\tau }_{0}\exp \frac{f-{f}_{0}}{T}$$

where *τ*_0_ is the minimum time for users to deal with a task. This response time is a very small quantity compared to the time span under our consideration.

According to Equation (1) and (3), we obtain the distribution of *τ* as,

4 $${\rm{\Psi }}(\tau )=P(f)|\frac{df}{d\tau }|=\frac{x{\tau }_{0}^{x}}{{\tau }^{x+1}}$$

The memory effect indicates that, when people are dealing with a sequence of tasks, there must be a reasonable order^29^. It limits large variations of *f* during state transitions. In order to involve this effect, we introduce $r(u)=r(\tau /{t}_{w})$ to denote the likelihood a user deals with a task with processing time *τ*. ${t}_{w}$ is a constant, and it will be eliminated in our following analysis. The likelihood function should satisfy the properties of $dr/du\mathrm{ < 0}$ and ${\mathrm{lim}}_{\tau \to \infty }r(\tau /{t}_{w}\mathrm{)=0}$. At any specific time point, let ${P}_{h}(\tau ,{t}_{w})$ denote the hitting probability that a user is dealing with a task with processing time *τ*. We have,

5 $${P}_{h}(\tau ,{t}_{w})\propto r(\frac{\tau }{{t}_{w}})\tau \Psi (\tau )$$

Hence, by re-organizing constant parameters, we have

6 $${P}_{h}(\tau ,{t}_{w})=Ar(\frac{\tau }{{t}_{w}})\frac{{t}_{w}^{x-1}}{{\tau }^{x}}$$

where *A* is the normalization constant.

As mentioned by the study of human interest dynamics^21^, the longer the time a person keeps doing something, the more probable he is to change his current interest. Hence, the longer the time *τ* a user is trapped into his former business, the more probable he is to see a certain message at present. Let ${p}_{m}$ denote the possibility that users transfer from doing other business to message browsing in microblogging platforms. To involve the above effect, the transition probability from the task with processing time *τ* at some point to message browsing in Weibo will be modified by multiplying the factor ${p}_{m}\exp (-t/\tau )$. Note that this factor and the memory effect factor *r*(*u*) do not affect the final form of the power-law relaxation equation, as long as they satisfy required properties^28^.

Let *N* denote the final number of retweets of an original message. At time point *t* after the publication of the message, let *n*(*t*) denotes the cumulative number of retweets at *t*. For those who have not retweeted the message yet, they will try $\langle \mathrm{1/}\tau \rangle dt$ times with transition probability ${p}_{m}$ and hitting probability ${P}_{h}(\tau )$. We assume that once a user decides to retweet a message, his action is instantaneous. $\langle \mathrm{1/}\tau \rangle$ denotes the average hopping frequency when users transfer from doing other business to tweet browsing. We calculate $\langle \mathrm{1/}\tau \rangle$ for all terms with *τ*. Let $r(\tau /{t}_{w})={t}_{w}/\tau$, we have,

7 $$\begin{array}{rcl}\langle \frac{1}{\tau }\rangle  & = & {\int }_{0}^{\infty }\frac{1}{\tau }Ar(\frac{\tau }{{t}_{w}})\frac{{t}_{w}^{x-1}}{{\tau }^{x}}{e}^{-\frac{t}{\tau }}d\tau /{\int }_{0}^{\infty }Ar(\frac{\tau }{{t}_{w}})\frac{{t}_{w}^{x-1}}{{\tau }^{x}}{e}^{-\frac{t}{\tau }}d\tau \\  & = & {\rm{\Gamma }}(x+\mathrm{1)}\frac{{t}_{w}^{x}}{{t}^{x+1}}/{\rm{\Gamma }}(x)\frac{{t}_{w}^{x}}{{t}^{x}}\\  & = & x/t\end{array}$$

Hence,

8 $$dn=(N-n(t))\cdot \langle \mathrm{1/}\tau \rangle dt\cdot {p}_{m}$$

9 $$\frac{dn}{N-n}={p}_{m}\frac{x}{t}dt$$

The general solution to the above differential equation is

10 $$n(t)=N-{C}_{0}{t}^{-x\cdot {p}_{m}}$$

where ${C}_{0}$ is a constant. Its value depends on the satisfaction of the boundary condition. We will discuss the values of parameters later. Equation (10) shows the cumulative dynamics of retweeting. It has the form of $c-a{t}^{-b}$.

### Model Fitting

We fit the power-function model $n(t)=c-a{t}^{-b}$ with our real-world tweet spreading data set.

We choose the top 5 most active influentials as the *root* users of their corresponding Direct Follower Networks. They are also the *authors* of the initialized tweets, which are the seeds of the chains of retweets that we trace. The identities of the influentials are diverse, including the official account of a news agency, two popular accounts that publish jokes and witticisms, a famous writer and a popular actress. There are altogether 2623 seed tweets initialized by these influentials.

**Figure 5:**
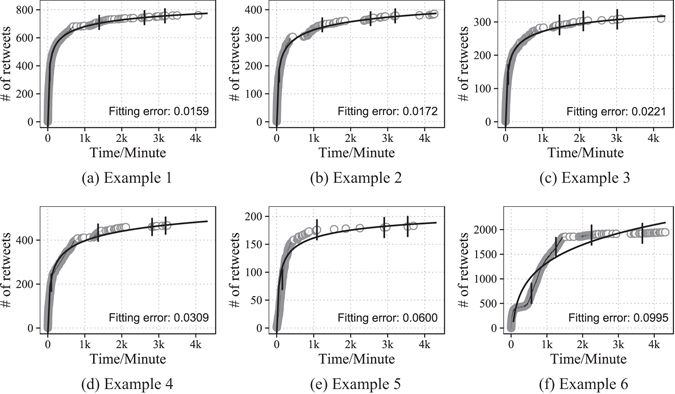
Model fitting examples with our real-world data set. The grey dots represent the cumulative numbers of retweets at different time points. And the black curves are the fitted models with the form of our power function. Error-bars are calculated based on the standard deviation of residuals.

**Figure 6:**
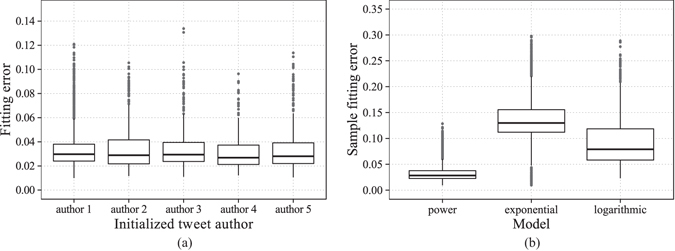
Model Fitting Results. (**a**) Distribution of fitting errors for the top 5 most active authors. (**b**) Comparison of fitting error distributions using different models.

We fit the 2623 chains of samples with the same model but different parameters (See Methods). The results show that for 2087 samples, the parameters *b* are in (0, 1), which account for 80% of all the samples. We choose normalized Root Mean Square Error (RMSE) as our performance measure. Some fitting examples are illustrated in Fig. 5. The figures show that those fitting results with errors lower than around 0.06 demonstrate the effectiveness of our model. The results with high errors are rare, and the reason of high errors is probably that there are several peaks of retweeting intensity during the whole process of spreading. We examine the samples with relatively large fitting errors, and discover that around 70% of the original tweets corresponding to these samples are published between 10 pm and 6am the next day. It is mainly the circadian rhythm^30^ that results in the spontaneous emergence of multi-peak retweeting patterns among direct retwitters. The distribution of all fitting errors is illustrated in Fig. 6(a). It is shown that the errors are less than 0.05 for around 85.9% of retweeting curves. The empirical data are well fitted by our model.

To further demonstrate the effectiveness of our power-function model, we fit the empirical data with two alternative forms, an exponential function (11) and a logarithmic function (12).

11 $$n(t)=c-a{e}^{-bt}$$

12 $$n(t)=c+a\,\mathrm{ln}(t+b)$$

The fitting method is identical to that for power-function fitting. Results are illustrated in Fig. 6(b). We find that the samples whose fitting errors are less that 0.05 account for 1.68% and 13.6% of total samples for exponential model and logarithmic model respectively. While this number is 85.9% for power-function model. Hence, we conclude that the derived power-function model outperforms both exponential and logarithmic models.

**Figure 7:**
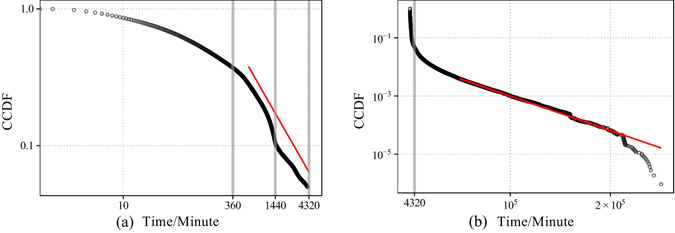
Complementary Cumulative Distribution Function (CCDF) of the waiting time for retweeting. (**a**) The distribution of *τ* within a relatively small time span. The tail is approximately a straight line. (**b**) The distribution of *τ* at a larger time span. The tail is likely to be exponential.

In order to show the distribution of the waiting time of retweeting *τ*, we plot the CCDF (Complementary Cumulative Distribution Function) of *τ* in Fig. 7. Within the time span under our consideration, which is 72 hours (4320 minutes), the tail of the distribution of *τ* shows approximately a straight line (the red line). However, at a relatively larger time span, the distribution indicates an exponential tail of the form $f(x)\sim \exp (-\theta x\mathrm{),\ }\theta \ge 0$, we adopt the maximum distance between the CCDF of the empirical distribution and the CCDF of the estimated exponential distribution as our criterion, which is similar to the Kolmogorov–Smirnov statistic. Then we estimate the exponent *θ*, and find the optimal value, which is $2.73\times {10}^{-5}$, approximately. The red line in Fig. 7(b) shows the fitting result. The criterion is approximately 0.01782, which is not large compared to 1.

Within a small time span, in which most spreading processes saturate, the distribution of *τ* is approximately power-law. This result is consistent with Equation (4).

The above results validate the power-function model of cumulative dynamics of independent spreading activities. The model implies a clear trend of saturation, and an aging effect, which means if the longer the time after the last retweeting activity happens, the longer the time it is to wait for the next retweeting. Model comparison demonstrates that the decay of the intensity of spreading is neither as fast as exponential, nor as slow as logarithmic.

### Model Validation

We conduct several empirical experiments to validate some key facts in our model, including

1.the relationship between parameter *a* and *c*.

2.the exponentially distributed landscape.

3.the connection between *b* and temperature.

These experiments are based on those samples with parameter *b* in (0, 1), which account for 80% of all samples.

**Figure 8:**
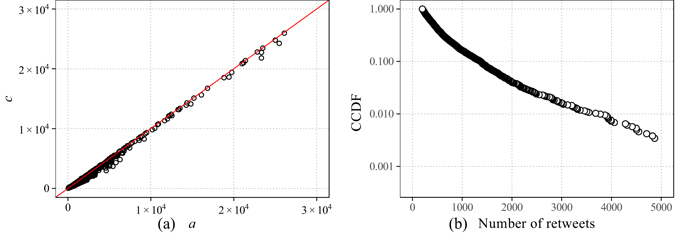
(**a**) The scatterplot for parameter *a* and *c*. (**b**) Complementary Cumulative Distribution Function (CCDF) of the number of retweets of original tweets. The approximate straight line on a single-log plot indicates the possibility of an exponential distribution.

To determine the value of the constant *C*_0_ in model (10), we need to investigate the satisfaction of the boundary condition $n(t\mathrm{=1)=0}$. If we precisely choose the minimal response time of retweeting as our base unit of time, it is possible to adjust the cumulative curves to meet the desired boundary condition. In this case, the boundary condition will hold and *a* equals *c*. However, there exist some technical limitations when collecting our data. And for the convenience of analysis, we choose one minute as our time unit. We calculate the ratio of the number of retweets within one minute. They are less than 5% for 96% of all samples. Hence, there are some small deviations in calculating the boundary condition. We draw a scatterplot of *a* and *c* in Fig. 8(a). It seems that *a* = c. However, a closer examination reveals that there are more points below the diagonal.

In addition, the bias in estimating the waiting time *τ* may affect the boundary condition. If we adopt the power function model of *τ* described by Equation (4), we can see from Fig. 7(a) that this power-law model is likely to overestimate the actual processing time near zero, which means that the cumulative number of retweets predicted by the model will grow slower than the actual number. A slower growth of the model indicates a relatively larger *a* in the form $n(t)=c-a{t}^{-b}$. And the curve generated by our model tends to shift to the right side of the actual curve. Due to this shifting, the model may produce negative values near zero. Hence, it is very possible that *a* > *c* when *t* = 1. And it is consistence with the results in Fig. 8(a). The effect of the above facts on the satisfaction of the boundary condition may be inevitable, but it is small. The above analysis demonstrates the consistency between the derived model and our empirical data.

The energy landscape of disordered systems, social systems and biological systems are well-studied^31–33^. The landscape of very low energies in REM is exponentially distributed. In our Weibo systems, the ground-state corresponds to users’ normal status in their daily activities. Once a user begins to browse and read tweets, he will be “trapped” into some humps created by the tweets he browsed. Most of the tweets will take up only a small amount of his time, but there are some tweets that will attract his attention. Hence, the landscape in Weibo systems is created by the tweets. Furthermore, the time one spends on each tweet is determined by the height of the hump. The popularity or attractiveness of a tweet is well-defined by how many times it has been retweeted during a relatively long period. Figure 8(b) shows the CCDF of the numbers of retweets of the original tweets in our data set. The figure indicates that the CCDF is approximately a straight line with a log-transformed *y*-axis. This results provide evidence that the energy landscape of Weibo systems is likely to be exponentially distributed.

Rate parameter *b* is crucial since it determines the overall shape of the growth of retweeting activities. The spin glass parameter *x* in Equation (1) in Reference 28, to which *b* corresponds, is temperature dependent. Since temperature represents the average kinetic energy of microscopic motions of particles, the physical model indicates that there is a connection between *b* and the average activeness of retwitters. The actual meaning that *b* reveals is related to the so-called *social temperature* concept, which is defined as the probability of one’s acceptance of others’ opinion^34, 35^.

**Figure 9:**
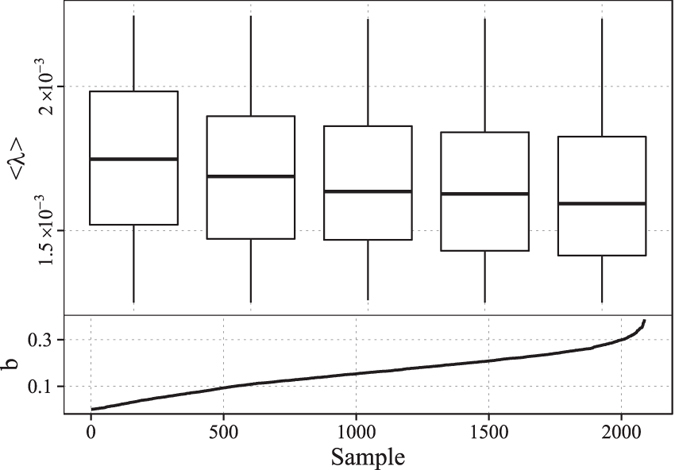
The variation of the relaxation exponent *b* and the distribution of the parameter *λ* of the exponential model fitted to groups of ${r}_{act}$.

Here, we introduce an indicator to represent the average activeness of retwitters. The number of a user’s followees Ω determines the amount of messages the user receives, and the number of messages *ω* the user retweets during a certain period determines to what extent the user approves others’ opinion. These two quantities are direct indicators of users’ activeness. Then we define the ratio ${r}_{act}=\omega /{\rm{\Omega }}$ as an indicator of retweeting activeness for each retwitter. If temperature rises, retwitters will be more active and the system will relax faster. For each retweeting sample, we calculate ${r}_{act}$ for every its retwitter. The distribution of ${r}_{act}$ for one sample is not fat-tailed. We then fit ${r}_{act}$ with exponential distribution and record the exponent *λ* for each sample. As shown in Fig. 9, as *b* increases, the distributions of *λ* in each group decrease. Since $\mathrm{1/}\lambda$ reflects the average activeness among the retwitters of a sample, the results demonstrate that as “social temperature” increases, the level of activeness among users increases too. This is consistent with our former analysis. The above empirical findings demonstrate the real significance that parameter *b* points to.

### Predictability

In order to demonstrate the function of our derived model, we carry out several prediction experiments, including two based on historical data and one based on microscopic data.

**Figure 10:**
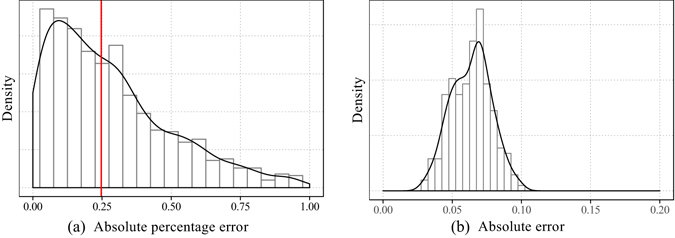
(**a**) Distribution of the absolute percentage errors of the prediction of number of saturated retweets. (**b**) The distribution of prediction error for parameter *b* by support vector regression.

At first, we try to predict the saturated number of retweets *N* by using historical data. We fit the curve of cumulative number of retweets by our power-function model at the 8th, 12th and 16th hour respectively after the publication of the original tweet for all our samples. Then we obtain 3 groups of parameters *a*, *b* and *c*. We use these 9 parameters as features to train a support vector regression model for the prediction of the number of retweets at the 72nd hour. We use a ten-fold cross validation to evaluate the accuracy of the prediction. The distribution of the absolute percentage errors between predicted number of retweets and the actual number is shown in Fig. 10(a). The red line indicates the median of the error, which is 24.7%. And the errors are lower than 40% for more than 70% samples.

In view of the difficulty of accurate prediction of the number of retweets for every single original message, the performance of the above prediction seems moderate.

The first experiment implies that an accurate qualitative classification model could be more practical. In order to examine the predictability of historical data, rather than the effect of network structure and message contents, we only select the samples that contain no multimedia contents and are published by a same author. The samples with the largest 200 *b* are labeled as positive, and the samples with the smallest 200 *b* are labeled as negative. We use the parameters fitted at the 8th, 12th and 16th hour after publication as features to train a support vector machine for classification. We adopt the ten-fold cross validation to evaluate the performance. The average accuracy, precision and recall are 85.25%, 90.60% and 79.50%, respectively. The results show that, by fitting historical data to our power-function model, we can obtain a high-performance classifier for predicting the magnitude of parameter *b*.

Next, we show the predictability by using microscopic data. In order to demonstrate the relationship between microscopic features and macroscopic measurements, by using a machine learning technique, we perform an experiment to predict the relaxation exponent *b*. The external magnetic field, which affects the relaxation of a spin glass system, corresponds to the “field” applied by the author and the original tweet in a Weibo system. We retrieve several microscopic features from authors’ profiles and the content of messages, and perform a support vector regression for the prediction of *b*. These features are detailed in Table 2. The features are extracted only from the profiles of the authors and the content of the original messages, since we believe that it is only the charm of the influentials and the topics in their messages that define the external magnetic field and then attract people to retweet.

**Table 2:** Generated Features for the prediction of *b*.

Feature	Description
Followee	No. of followees of an author
Follower	No. of followers/fans of an author
Activeness	No. of messages an author posted in the past
VIP	VIP certification of an author
URL	No. of URLs in an original message
Hashtag	No. of hashtags in an original message
Mention	No. of mentions in an original message (@username)
Multimedia	No. of videos and musics in an original message
Retweets	No. of retweets of the original message

We perform a ten-fold cross-validation to test the performance of our prediction. The absolute errors are shown in Fig. 10(b). Since $b\in \mathrm{(0,1)}$, the mean value of the absolute error, which is 0.064, is not large. This experiment shows that the differences in *b* are partly due to the effect of the differences in the identities of authors and also the content of messages. The gap between microscopic mechanisms and macroscopic phenomena can be bridged in this way.

The above experiments demonstrate, to some extent, the predictive capability of our model.

### An Application

Here we demonstrate a possible application of our model, which is the examination of whether or not a spreading process is approaching to saturation.

If the parameter *b* in the model of $n(t)=c-a{t}^{-b}$ is positive, then *n* → *c* as $t\to \infty$, which means the spreading process is approaching to saturation. If a spreading process approaches to saturation, the model parameter *b* estimated by the saturated curve will be greater than 0. However, at the early stage of a spreading, *n* grows in a divergent way. The model parameters *a* and *b* will be both negative. Hence, if we estimate the model parameters with truncated data according to the time order, the value of parameter *b* will monotonically increase from negative to positive.

**Figure11:**
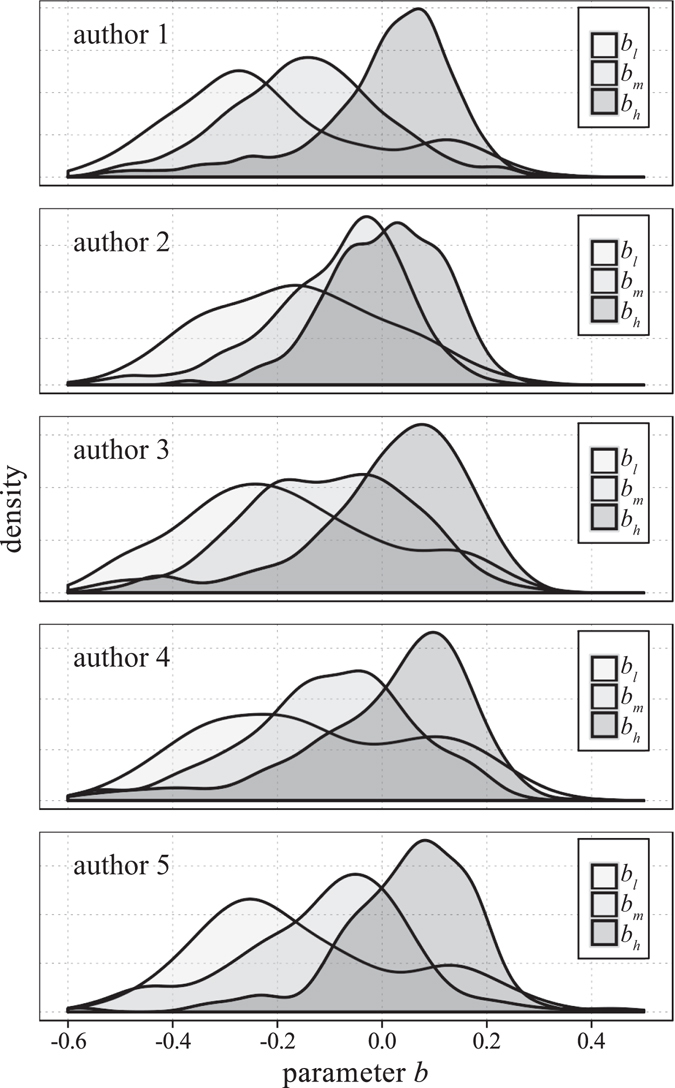
Variation of the distributions of *b*_*l*_, *b*_*m*_ and *b*_*h*_.

By the estimation of the value of model parameter *b*, we are allowed to examine if a spreading process reaches saturation or not. We conduct a simple experiment to explore the application potential of our model. We truncate our data by the 4th, 6th, …, 82th hour and fit our power-function model for parameters *a*, *b* and *c*, respectively. We record the change of the values of *b* at different hours. Then we record the time points *t*_*l*_, *t*_*m*_ and *t*_*h*_ at which the cumulative number of retweets reaches 65%, 80% and 95% of the saturated number of retweets. According to the change of *b*, we record the values ${b}_{l}$, *b*_*m*_ and *b*_*h*_ that correspond to time points *t*_*l*_, *t*_*m*_ and *t*_*h*_. We calculate all ${b}_{l}$, *b*_*m*_ and ${b}_{h}$ for each tweet spreading sample. The distributions of ${b}_{l}$, *b*_*m*_ and *b*_*h*_ for the 5 influentials are illustrated in Fig. 11.

The results show that for all 5 influentials, the peak values of the distributions of *b* increase. And when the number of retweets reaches 95% of final saturated number, all peak values of the distributions of *b* exceed zero. We could utilize this feature to roughly identify whether or not a retweeting process approaches to saturation at present. Specifically, we could monitor and record the growth of cumulative number of retweets dynamically. Then we fit the recorded data with our power-function model at a certain time point. We learn and choose a threshold larger than zero based on historical experiences. When the value of parameter *b* in the fitted model exceeds the threshold, we could make our decision that the spreading process reaches saturation approximately. Accurate identification of the saturation of spreading will be beneficial to the scheduling of intervention, such as influence maximization and advertising promotion using microblogging services.

## Discussion

Both our model and data show clear saturation patterns of independent retweeting behaviour. The reasons may be a mixture of the fading of interest and the limit of the size of personal devices. Microblogging is a platform for high-speed information exchange. People will quickly lose interest on one topic and shift to another. In addition, because of the size limit of screens on personal devices and the speed of newly generated content, people will miss large amounts of information easily and will be unlikely to take efforts for digging old messages. The combined effect of these factors results in the saturation of spreading.

A possible explanation to the exponentially distributed landscape could be given based on the Boltzmann distribution, which characterizes the distribution of particles on different energy states of isolated systems that are in thermal equilibrium. Similar to thermodynamic systems, retwitters in our systems are reasonably assumed to be separable. And their interest orientations may correspond to particles’ degeneracy states. The energy of a retwitter may represent his activeness in the sense of retweeting. The states of retwitters are only associated to their energy property. Retwitters could be distributed over different energy states. According to the principle of equal *a priori* probabilities, the distribution with the largest number of micro-states occurs with the highest probability. This *Most Probable Distribution* leads to the exponential form of the Boltzmann Distribution.

Among our fitting results, we find that most retweeting samples with relatively large fitting errors show multi-peak patterns in their intensity curves of retweeting. The main reason is the circadian rhythm in people’s daily life. When an influential publishes a tweet in daytime, most active retwitters have enough time to response to the message. While if the message is initialized around people’s sleeping period, a portion of the retwitters are inactive at that moment. It looks like retweeting activities are paused at night and restored in the morning. This leads to multi-peak patterns and relatively large fitting errors. We plan to take into account this effect in our future work.

Parameter *b* changes if we choose different lengths of time intervals for fitting. The reason is that as the original message is getting older, due to the limit of screen sizes, the number of users who will see the message is getting smaller. Retweeting happens when two conditions hold: first, users must have the willingness to retweet a certain message; second, users must have the chances to see the message. The number of users who are willing to retweet a certain message may be relatively stable. However, the number of users who will see the message decreases after its publication, unless other influentials retweet it during the whole spreading process. Users will pay more effort for searching if a message is getting older.

The significance of our work is two-fold. First, this work demonstrates that applying physical theories to research fields outside physics, such as social sciences and economics^24, 36–38^, is of great significance. It is true that data fitting methods are widely used to summarize the relationships among variables and to infer values. However, when different scenarios are considered, we need to repeat fitting procedures in a possible high-dimensional parameter space. In addition, the actual meanings of the fitted parameters could be ambiguous. The analogy with physical systems helps us derive a model that explains the formation of the data with microscopic mechanisms and meaningful parameters. Once the changes of parameters are measured, the model can be applied in other scenarios. Second, our work indicates that some invariable rules should be taken into consideration during the prediction of information spreading. Traditionally, various features retrieved from users, message text and network structures are adopted in well-tuned models for prediction. However, we find that our derived model with a unified form governs the independent retweeting behaviours with different authors and contents in our data set. And some features, such as memory effect, seem to be common to different retweeting activities. Hence, some common rules, which are ignored before, may be useful for prediction.

## Materials and Methods

### Data Description

We obtain two data sets from Sina Weibo for our study. The message data set consists of more than 69 million tweets/retweets from Sina Weibo. There are 6 properties associated with each tweet, (1) message text, (2) original publisher’s ID and nickname, (3) timestamp, (4) number of retweets, (5) number of comments, (6) a flag to indicate that the tweet is either originally published or retweeted. The link data set consists of 3.7 billion directed links among 80 million users.

### Model Fitting Method

For each of the 2623 seeds, all of its retweets are collected. We use regular expressions to parse the retweet tag in the text of retweets. Then a chain of retwitters of the root author could be retrieved chronologically for each branch of retweets of the initialized tweet. In this way, we could locate the retwitters who directly retweet the root user’s tweets, rather than indirectly retweet it from other retwitters.

For each initialized tweet, we plot the curve of its cumulative number of retweets with respect to time. We intend to fit these curves with our power function model. We choose Root Mean Square Error (RMSE) as our objective error function to measure the goodness that real-world data are observed to fit our model. In addition, in order to make the fitting errors of different samples comparable, we divide RMSEs by the maximum/saturated numbers of retweets of the corresponding initialized tweets. This division has no effect on parameter optimization, and it provides a proper way of evaluating the goodness of fitting. Equation (13) defines our objective function.

13 $${E}_{obj}=\frac{1}{{M}_{N}}\sqrt{\frac{1}{N}\sum _{t\mathrm{=1}}^{N}{({M}_{t}-{\hat{M}}_{t})}^{2}}$$

where *M*_*t*_ is the cumulative number of retweets in empirical data at time *t*, ${\hat{M}}_{t}$ is the model output at time *t*. The time granularity is set to 1 minute, and the maximum of *t* is set to 72 hours since most spreading processes will saturate in DFNs at the end of the third day after the initialization of the seed tweets. Then *N* is 4,320. And *M*_*N*_ is the saturated number of retweets, i.e., the maximum retweeting number.

We assume that the objective function to be minimized is $F(a,b,c)$ with respect to parameters $a$, $b$ and $c$. The first order total derivative of $F$ with respect to $b$ is

14 $$\frac{dF}{db}=\frac{\partial F}{\partial b}+\frac{\partial F}{\partial a}\frac{\partial a}{\partial b}+\frac{\partial F}{\partial c}\frac{\partial c}{\partial b}$$

It is difficult to calculate the first order and second order total derivatives of the error function with respect to parameter *b*, since *b* occurs as the exponent of variable *t*. However, if we set $\partial F/\partial a\mathrm{=0}$ and $\partial F/\partial c\mathrm{=0}$, $dF/db$ equals to $\partial F/\partial b$. Parameters *a* and $c$ are represented by *b*. Hence, in order to avoid the expensiveness, we adopt a Quasi-Newton method to find the optimal parameter set. The convexity of *F* with respect to *b* is difficult to prove. However, the searching algorithm yields good results.
